# Map of Prolonged Electrogram Duration to Guide Atrial Substrate Ablation for Atrial Fibrillation Recurrence Following Durable Pulmonary Vein Isolation

**DOI:** 10.19102/icrm.2021.120124S

**Published:** 2021-01-15

**Authors:** Pietro Rossi, Filippo Maria Cauti, Marco Polselli, Stefano Bianchi

**Affiliations:** ^1^Cardiology Division, Arrhythmology Unit, S. Giovanni Calibita Hospital, Isola Tiberina, Rome, Italy

**Keywords:** Complex fractionated atrial electrograms, electrogram duration, functional substrate, persistent atrial fibrillation, voltage map

Pulmonary vein isolation (PVI) is considered an appropriate therapeutic strategy for persistent atrial fibrillation (AF) (PAF) even if less effective than that for paroxysmal AF. The recurrence of PAF depends upon the extent, localization, and degree of atrial tissue damage. Performing an atrial tissue substrate study is highly desirable in patients with PAF, especially in cases of recurrence after PVI, to improve the ablation outcome.

We describe a patient (55 years old) affected by highly symptomatic PAF who underwent PVI. After four months, despite antiarrhythmic drug therapy, the patient experienced PAF recurrence and a second intervention was planned. During the intervention, an external electric cardioversion was firstly performed to restore sinus rhythm (SR). Voltage mapping (0.1–0.5 mV) during SR demonstrated no areas of low voltage.

The procedure was performed using the EnSite Precision™ cardiac mapping system and the multipolar diagnostic Advisor™ HD Grid Mapping Catheter, Sensor Enabled™ **(Video 1)**. Voltage and atrial electrogram duration maps (AEDUM method) were created by using the EnSite Precision™ AutoMap™ mapping tool.

Bipole electrogram (EGM) durations were measured using the Ensite Precision™ TurboMap™ tool, which allowed us to review SR maps and calculate the duration of each point as the temporal distance (ms) between the first and last deflections of each endocardial EGM. A high-density wave configuration reduced the limitation of angle variance between the bipole pair and wavefront propagation **(Figure 1)**. A cutoff of 45 ms for EGM duration was applied based on our previous experience of left atrium mapping in subjects without a history of AF (unpublished data). The area with slow and inhomogeneous conduction was ablated, applying a power level of 40 W, with guidance provided by the lesion index. The patient remained free from recurrence after eight months of clinical follow-up.

The proposed approach could allow operators to tailor the ablation scheme for each patient depending on the location and extension of areas with slow conduction. In this case, the roof and the anterior regions were involved instead of the posterior wall.

## Figures and Tables

**Figure 1:**
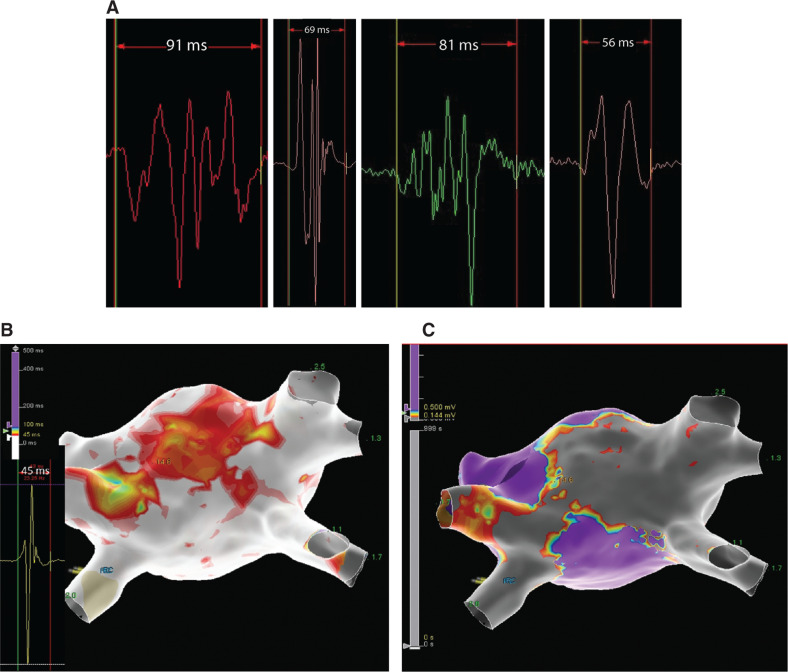
**A:** Representative prolonged EGMs recorded in the roof and anterior region of the left atrium during SR using the Advisor™ HD Grid diagnostic catheter. **B:** Three-dimensional left atrium map with an area of prolonged EGMs as a marker of slow and inhomogeneous conduction. **C:** Voltage map view indicating the extension of the atrial ablation involving the red area with conduction abnormalities shown in **B**.

**Video 1.** 

